# CAFs-derived lactate enhances the cancer stemness through inhibiting the MST1 ubiquitination degradation in OSCC

**DOI:** 10.1186/s13578-024-01329-y

**Published:** 2024-11-27

**Authors:** Shuzhen Zhang, Jingjing Wang, Yang Chen, Weilian Liang, Hanzhe Liu, Ruixue Du, Yunqing Sun, Chuanyu Hu, Zhengjun Shang

**Affiliations:** 1 State Key Laboratory of Oral & Maxillofacial Reconstruction and Regeneration, Key Laboratory of Oral Biomedicine Ministry of Education, Hubei Key Laboratory of Stomatology, School & Hospital of Stomatology, Wuhan University, Wuhan, China; 2 Department of Oral and Maxillofacial-Head and Neck Oncology, School & Hospital of Stomatology, Wuhan University, Wuhan, 430079 China; 3 Department of Oral and Maxillofacial Surgery, School & Hospital of Stomatology, Wuhan University, Wuhan, 430079 China; 4 Department of Stomatology, Tongji Hospital, Tongji Medical College, Huazhong University of Science and Technology, Wuhan, 430030 China; 5 School of Stomatology, Tongji Medical College, Huazhong University of Science and Technology, Wuhan, China; 6 Hubei Province Key Laboratory of Oral and Maxillofacial Development and Regeneration, Wuhan, China; 7 Central Department School & Hospital of Stomatology, Wuhan University, Wuhan, 430022 China; 8 Day Surgery Center, School and Hospital of Stomatology, Wuhan University, Wuhan, China

**Keywords:** Lactate, Cancer stem cells, DLG5, Hippo pathway, Ubiquitination

## Abstract

**Background:**

Cancer-associated fibroblasts (CAFs), a predominant stromal cell type in the tumor microenvironment, significantly affect the progression of oral squamous cell carcinoma (OSCC).

**Results:**

The specific mechanisms through which CAFs influence the cancer stem cell phenotype in OSCC are not fully understood. This study explored the effects of lactic acid produced by CAFs on the cancer stem cells (CSCs) phenotype of OSCC cells. Our results demonstrated that CAFs exhibit increased glycolysis and lactic acid production. Lactic acid treatment enhances CSCs-related markers expression, sphere formation, and clonogenic ability of OSCC cells. RNA sequencing revealed that lactic acid treatment elevates Discs Large Homolog 5 (DLG5) expression and markedly affects the Hippo pathway. Further investigation revealed that DLG5 mediates the effects of lactic acid on the CSCs phenotype. DLG5 knockdown results in elevated expression of E3 ubiquitin ligase Cullin 3, which can promote the ubiquitination and degradation of MST1, but the expression of phosphorylated MST1 remains unchanged. This leads to enhanced binding of phosphorylated MST1 to YAP1, increasing YAP1 phosphorylation and activating the Hippo pathway.

**Conclusion:**

Collectively, our findings suggest that lactic acid from CAFs promotes the CSCs phenotype in OSCC through the DLG5/CUL3/MST1 axis. Therefore, targeting lactic acid exchange between CAFs and tumor cells may provide a novel therapeutic approach to suppress the CSCs phenotype in OSCC.

**Supplementary Information:**

The online version contains supplementary material available at 10.1186/s13578-024-01329-y.

## Introduction

Oral squamous cell carcinoma (OSCC) is the most prevalent malignant tumor in the head and neck region. In 2022, there were 389,846 new cases of lip and oral cancer worldwide, resulting in 188,438 deaths (https://gco.iarc.fr), the five-year survival rate of OSCC patients is less than 50% [1, 2]. Emerging evidence indicates that the tumor microenvironment plays a pivotal role in cancer initiation and progression, highlighting the inadequacy of therapies solely targeting cancer cells [3, 4]. Cancer-associated fibroblasts (CAFs) are the primary stromal cells within the tumor microenvironment that influence cancer progression through direct and indirect mechanisms, including cell‒cell interactions, release of soluble factors, and remodeling of the extracellular matrix [5–7]. Previous investigations have demonstrated that tumor cells induce proangiogenic changes in CAFs via the SOCS1/JAK2/STAT3 signaling pathway [8]. Additionally, CAFs undergo metabolic reprogramming through the p-ERK1/2 pathway [9], resulting in the degradation of CAV1 and increased production of lactic acid to support the growth of cancer cells, thereby promoting the progression of OSCC [10]. Lactic acid, previously considered a metabolic waste product, is now widely recognized as both a substrate for energy metabolism and a molecule that influences epigenetic regulation [11]. In the tumor microenvironment (TME), hypoxic cells generate a significant amount of lactic acid through Warburg metabolism, which is then transferred to neighboring cells for oxidative phosphorylation [12]. This lactic acid transfer enhances the redistribution and efficient utilization of energy substrates to meet the rapid growth demands of tumor cells [12–14]. Furthermore, lactic acid can directly inhibit signaling pathways by modifying histones, thereby playing a crucial role in tumor immunity [15–17]. The level of lactic acid in the TME is strongly associated with cancer aggressiveness and lower survival rates [18]. Cancer stem cells (CSCs), also referred to as tumor-initiating cells, represent a small subpopulation of tumor cells with robust self-renewal capabilities and potential for multilineage differentiation. Our preliminary studies indicate that under adverse conditions such as starvation, hypoxia, and cisplatin treatment, the level of autophagy in tumor cells increases, leading to their transition to a CSCs phenotype through the degradation of FOXO3 [19]. Additionally, we discovered that lactic acid produced by CAFs promotes the development of the CSCs phenotype in OSCC [20]. However, the precise mechanisms by which lactic acid regulates the CSCs phenotype remain poorly understood, necessitating further research.

The Hippo pathway, a highly conserved cellular signaling cascade consisting of MST1/2, LATS1/2, and YAP/TAZ, is responsible for regulating cell proliferation and apoptosis to control cell differentiation and organ size [21, 22]. Wang et al. conducted a comprehensive molecular characterization of 19 core Hippo pathway genes using multidimensional 'omics' data from The Cancer Genome Atlas. They analyzed 9,125 tumor samples from 33 cancer types and demonstrated that alterations in the Hippo cascade and its effectors YAP and TAZ are significant drivers of carcinogenesis, including head and neck squamous cell carcinoma. They also highlighted the crucial role of Hippo pathway dysregulation in maintaining the CSCs phenotype [23]. However, it is still unclear whether the Hippo pathway is involved in lactic acid-mediated CSCs phenotypic transformation.

Discs Large Homolog 5 (DLG5), a key member of the membrane-associated guanylate kinase family, is a scaffolding protein involved in various cellular processes, such as maintaining epithelial cell polarity, cell adhesion, and progenitor cell division and transmitting extracellular signals to the cell membrane and cytoskeleton [24]. Low expression of DLG5 has been positively correlated with the development and progression of several cancers, including lung squamous cell carcinoma [25], breast cancer [26], liver cancer [27, 28], prostate cancer [29], and bladder cancer [30]. However, two independent studies on glioblastoma reported conflicting effects of DLG5 on tumors. Su et al. reported that overexpression of DLG5 activates the Hippo pathway by increasing YAP phosphorylation, thereby inhibiting the malignant behavior of glioblastoma cells [31]. In contrast, Kundu et al. reported that high expression of DLG5 in glioblastoma maintains the self-renewal and invasiveness of CSCs through regulation of the Sonic Hedgehog signaling pathway, promoting tumor progression [32]. Therefore, further exploration is needed to determine whether lactic acid can influence the CSCs phenotype through the regulation of DLG5 expression and whether the Hippo pathway is involved.

In this study, we investigated the role of CAFs in the context of OSCC. We discovered that CAFs exhibit a high level of glycolysis and produce lactic acid. Moreover, we found that lactic acid enhances the CSCs-like phenotype in OSCC. We elucidated the specific molecular mechanism through which lactic acid regulates the CSCs phenotype, which involves the DLG5/HIPPO/YAP axis. Our findings provide new insights and potential strategies for targeted therapies of CSCs in OSCC.

## Methods and materials

Additional methodologies are detailed in the Supporting Information.

### Cell culture

Patient-derived para-cancer fibroblasts (PFs) and cancer-associated fibroblasts (CAFs) were obtained from tumor samples and adjacent non-tumor tissues of OSCC patients at the Stomatological Hospital of Wuhan University. Our research has been approved by the Ethical Committee on Animal Experiments of the Animal Care Committee of Wuhan University (IRB-ID: 2021A18). NIH-3T3 cells were induced to obtain murine-derived CAFs using 5ng/ml TGF-β. CAFs were cultured with DMEM (HyClone, USA) containing 10% FBS (Gibco, USA).

SCC7 was purchased from Otwo Biotech (Guangzhou, China) and cultured in RPMI 1640 medium (HyClone, USA) containing 10% FBS, while CAL27 was purchased from Cell Bank and Stem Cell Bank of Chinese Academy of Sciences (Shanghai, China) and cultured in DMEM medium containing 10% FBS. All cells were cultured in a 37℃ incubator with 5% CO_2_.

### Western blotting

Whole cell lysates (WCL) were prepared on ice using a mixture of RIPA lysis buffer (P0013K, Beyotime) and protease and phosphatase inhibitors (minitab, Fisher Scientific). The protein concentration of the sample was determined by BCA protein assay kit (P0012S, Beyotime), and separate equal amounts of protein using 10% SDS-PAGE. The imprinting was sealed with 5% skim milk, incubated with the primary antibody at 4℃ overnight, and then incubated with the coupled secondary antibody for 1h. The imprinting was detected with the ECL kit (P0018FS, Beyotime). ACT (10,366–1-AP), PKM2 (15,822–1-AP), CD44 (10,366–1-AP), CD133 (20,874–1-AP), ALDH1A1 (12,129–1-AP), NANOG (10,366–1-AP), BMI1 (12,129–1-AP), MST1 (22,245–1-AP), p-MST1 (80,093-J-RR), YAP1 (66,900–1-IG), OCT4 (10,366–1-AP), MCT4 (20,899–1-AP) antibodies were purchased from Proteintech (Chicago, USA), CUL3 (2759s) was purchased from Cell Signaling Technology (MA, USA), DLG5 (A302-301A) was purchased from ThermoFisher (Waltham, USA), p-YAP1 (ab254343), FAP (ab53066), α-SMA (ab7817), PFKFB3 (ab181861) were purchased from Abcam (Cambridge, United Kingdom). Visualization was performed using the protein imaging System Odyssey System (Li-Cor Biosciences) and density analysis was analyzed using ImageJ software (v1.50i, National Institutes of Health, Bethesda, MD, USA).

### Extracellular Acidification Rate

Extracellular Acidification Rate (ECAR) was measured in real time using a ECAR Fluorometric Assay Kit (E-BC-F069, Elabscience, USA). PFs and CAFs cells (with an initial density of 4 × 10^4^ cells/well) were seeded in microwell plates one day before the test. After incubation overnight, the medium was changed and the cells were treated with 1mM glucose, 1 μM oligomycin, and 50 mM 2-deoxy-d-glucose at designated time points. ECAR values were measured using the Seahorse Bioscience XF24 extracellular flux analyzer (XFe24, USA) to assess the glycolytic capacity of cells.

### Real-time PCR assay

Total RNA was extracted using TRIzol (Tokyo Takara, Japan). 1μg RNA was reverse-transcribed to cDNA template using PrimeScript RT kit. RT-PCR was performed in the QuantStudio 6 Flex qPCR system. Gene changes were calculated by comparison C. The primer sequence used were mentioned in Table S1 (Supplemental file 3).

### Lactic acid content determination

Collect NFs, CAFs and NIH-3T3 cells before and after induction of TGF-β, the lactate content was measured using the Lactic Acid Assay Kit (A019-2–1, Jiancheng, Nanjing, China) according to the manufacturer’s instructions.

### CCK8 assay

5 × 10^3^ SCC7 and CAL27 cells were plated in 96‐well plates and incubated with lactate 5.6 mM, 5.1 mM respectively for 24, 48 and 72 h. Then according to the manufacturer’s instructions, cells were cultivated with Cell Counting Kit‐8 (CCK‐8) solution (Beyotime, Wuhan, China) for 2 h. The proliferation capacity of the cell is determined by the optical density (OD) at 450 nm.

### Immunofluorescence

The cells were fixed on 24-well plates with 4% paraformaldehyde, closed with 5% bovine serum albumin (BSA) for 1 h, then incubated with primary antibody at 4 ℃ overnight, and stained with fluorescent secondary antibody at room temperature for 1h away from light. Images were obtained with an inverted fluorescence microscope and a confocal microscope (Olympus FV1200, Japan).

### The sphere forming assay

The 12-well plates were pretreated with polyHEMA (10g/L in 95% ethanol; Millipore, Sigma) to prepare low adhesion dishes. Preheating standard CSCs medium at 37 ℃: DMEM/F12 (Life Technologies, Waltham, MA) with 2% B27 supplement, 20ng/mL human recombinant epidermal growth factor (PeproTech), and 20ng/mL alkaline fibroblast growth factor (PeproTech) added. The cells were resuspended on standard CSCs medium (1000 cells/well). After 10 days, inverted microscopy was used to count balls with a diameter of 50 μm or more.

### Colony formation assay

Single-cell suspension was prepared, and thousand cells were seeded in each well of the six-well plate for 2 weeks. Afterward, the colonies were fixed and stained with crystal violet. The number of colonies was calculated under an optical microscope and colony area was assessed using Image-Pro Plus.

### Sample collection

For tissue microassay, 7 OSCC and 7 normal oral mucosa specimens were collected from the Stomatological Hospital of Wuhan University. Written informed consents were obtained from all the participants. The clinical and pathological characteristics of all patients were summarized in the supplemental file 4 and 5. Our research has been approved by the Ethics Committee of School and Hospital of Stomatology, Wuhan University (2022A05).

### Co-inmunoprecipitation

The cells were lysed at 4 °C with 1ml of IP cleavage buffer (P0013, Beyotime) containing a protease inhibitor for 15 min. The WCL were collected and centrifuged at 10,000*g* at 4 °C for 10 min. Then 1ml supernatant was incubated with 1 μg anti-CUL3 antibody (2759s, CST) and anti-MST1 antibody (22,245–1-AP, proteintech) overnight at 4 °C followed by addition of 20 μl Pierce™ protein A/G Plus agarice (20,423, Thermo) and incubated overnight at 4 °C. Then, the samples were washed four times with an immunoprecipitation cracking buffer and the immune complex was eluted with a sample buffer containing 1% SDS at 95 °C for five minutes. The immunoprecipitated protein and total lysate were separated by SDS-PAGE followed by Western blot analysis.

### Lentiviral transfection and RNA interference

First, the transfer plasmid pLV3-U6-DLG5-shRNA-Neo or pLV3-U6-CUL3-shRNA-Puro, packaging plasmid psPAX2, and envelope plasmid VSV-G were used to construct lentiviruses in 293T cells. Then the lentiviruses in the 293T supernatant were collected and used to infect cancer cells. Following a 48h period of infection, cell lines were screened using antibiotics to obtain stably transfected cell lines. For the co-infection cell lines, neomycin (Biosharp, BL1303A, 400μg/mL) was used to obtain DLG5 knockdown stable cell lines following shDLG5 lentiviruses infection. Then the DLG5 knockdown cells were infected with shCUL3 lentiviruses, and puromycin (ST551, Beyotime, 2μg/mL) was used to obtain double-knockdown stable cell lines. Western blot assay was used to detect gene knock-out inefficiency.

For RNA interference, we purchased two siRNAs targeting MCT4 and control RNAs from Genepharm (Suzhou, China). The siRNA sequences used are listed in Table S2 (Supplemental file 3). When the cell density reached approximately 70%-90%, the siRNA-Lipo3000 mixture was added to the cell culture dishes with FBS-free medium, and after 48–72 h of incubation, the cell samples were collected.

### Cell co-culture

Firstly, we purchased a six-well plate (Corning, 3412) dedicated for cell co-culture, inoculated about 3 × 10^5^ tumor cells in the lower chamber, added diluted stromal gel in the upper chamber first, and subsequently inoculated about 3 × 10^4^ CAFs in the upper chamber, and harvested the tumor cell samples after 48h of co-culture.

### EdU experiment

The EdU test was performed using the BeyoClick™ EDU-594 cell proliferation assay kit (Beyotime, Wuhan, China). SCC7 and CAL27 cells (1 × 10^4^) were uniformly inoculated into 12-well culture plates, cultured with EdU reagent for 2 h, and fixed the cells with 4% polyformaldehyde (Servicebio, Wuhan, China). Then, according to the manufacturer's instructions, add Click reaction solution and incubate at room temperature in the dark for 30 min, and then use Hoechst33342 reaction solution and incubate at room temperature in the dark for 30 min. Finally, the images were observed and obtained with a fluorescence microscope (Biozero BZ-8000, Keyence, Osaka, Japan).

### CHX chase assay

SCC7 and CAL27, as well as cells transfected with Sh-DLG5 were noculated into 12-well plates. When the cell density reached 70–80%, cells were treated with 100 μg/ml cycloheximide (CHX, Thermo Fisher Scientific, Waltham, MA) for 0, 2, 4, and 8h and harvested for western blot assay.

### Mouse xenografts

In vivo studies were conducted in accordance with the regulations of the Ethical Committee on Animal Experiments of the Animal Care Committee of Wuhan University (S0792203066). 5-week-old female BALB/c nude mice were obtained from Beijing Vital River Laboratory Animal Technology Co., Ltd. (Beijing, China) and housed in a specific pathogen-free (SPF) facility. The light/dark cycle was set at 12 h each. The mice were randomly divided into three groups: one group received injections of CAL27 cells transfected with an empty vector lentivirus (sh-SCR), another group received lactate (HY-B2227, MCE) culture treatment (sh-SCR + Lactate), and the third group received DLG5 lentivirus knockout followed by lactic acid culture treatment (Lactate + shDLG5). Tumor length and width were measured using a caliper every 5 days, and tumor volume was calculated using the formula: (width^2^ × length)/2. Throughout the experiment, the health status and mortality of the mice were monitored. Upon reaching the experimental endpoint, mouse tumors were harvested, resected, weighed, photographed, and evaluated.

### Statistical analysis

Statistical analysis was performed using GraphPad Prism 8 software (GraphPad 8.0, San Diego, CA, USA). Two-tailed unpaired Student’s t test and one-way analysis of variance were used to compare the differences between two independent groups, and Dunnett's multiple comparison test was used to analyze more than two groups. Results were expressed as mean ± standard deviation, with p < 0.05 considered statistically significant.

## Results

### CAFs exhibit increased glycolytic activity and lactate production

**Fig. 1:**
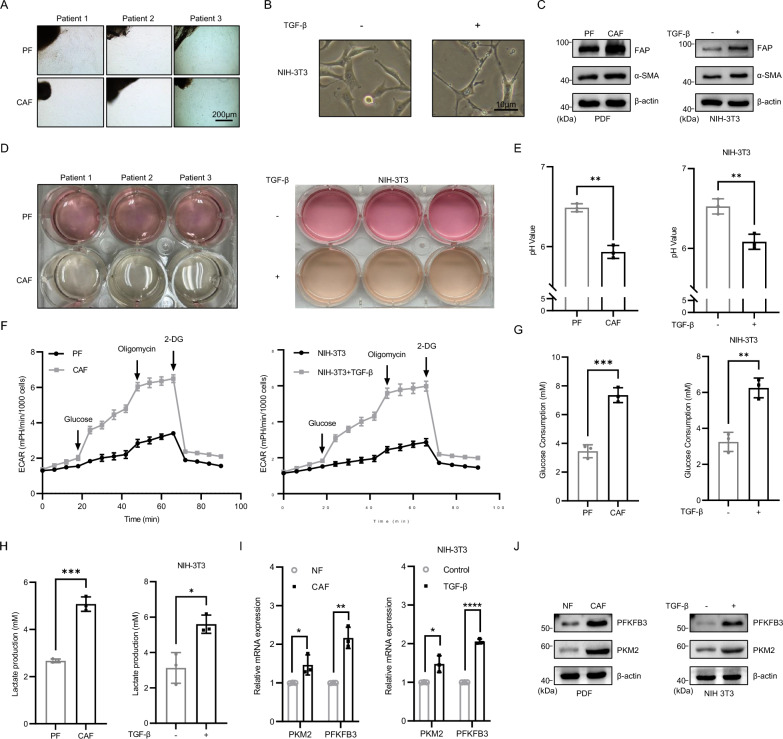
CAFs exhibit increased glycolytic activity and lactate production. **A** Extract bright field images of primary CAFs and PFs from three pairs of patients. Scale bars, 200 μm. **B** Extracting bright field diagrams of TGF-β-treated (10ng/ml) NIH-3T3. Scale bars, 10 μm. **C** Western blot results of CAFs-related markers were used to prove that the obtained cells were CAFs. β-actin was used as a control. **D**, **E** Clear flow chart of CAF and PF, as well as NIH-3T3 and its induced cells culture medium after the same cultivation time, as well as statistical chart of the pH value of the culture medium. Student’s t-test. **F** ECAR experiment was used to detect the release rate of acidic metabolites produced by cells. **G** Glucose consumption experiment was used to detect the rate of glucose consumption and uptake by cells. **H** Detecting the lactate secretion levels of CAFs, PFs, NIH-3T3 and their induced cells separately. **I**, **J** RT-qPCR and western blot results of glycolysis-related markers. β-actin and GAPDH were used as a control. Independent experiments (in vitro) were performed in triplicate. Data are presented as mean ± SD. *P < 0.05, **P < 0.01, ***P < 0.001, ****P < 0.0001

To investigate the metabolic characteristics of CAFs in OSCC tissues, CAFs and paired fibroblasts (PFs) were isolated from tumor samples and adjacent nontumor tissues of OSCC patients (Fig. 1A). In addition, CAFs were generated from mice by inducing NIH-3T3 cells with transforming growth factor-beta (TGF-β). Under bright field microscopy, PFs and untreated NIH-3T3 cells appeared flat, spindle-shaped, and small in size, whereas CAFs and TGF-β-treated NIH-3T3 cells were elongated, spindle-shaped, and enlarged (Fig. 1B). Fibroblast activation protein (FAP) and alpha-smooth muscle actin (α-SMA) are key markers of CAFs, and immunoblotting results indicated increased expression of FAP and α-SMA in both human and TGF-β-induced murine CAFs (Fig. 1C). These findings confirm the successful derivation of CAFs from human and murine sources. Importantly, culture media inoculated with an equivalent number of normal fibroblasts and CAFs turned yellow rapidly in CAF cultures, whereas PF cultures maintained a pink hue for a longer period (Fig. 1D), suggesting greater lactate production by CAFs. Consequently, the pH of the culture supernatants was measured, revealing that the pH values for human and murine CAFs were 5.93 and 6.08, respectively, compared to 6.48 and 6.52 (Fig. 1E), respectively, for PFs. Further analyses of glucose consumption, lactate production, and the extracellular acidification rate (ECAR) demonstrated that CAFs consumed glucose more rapidly and produced significantly more lactate (Fig. 1F–H). Subsequent PCR and immunoblotting assays to evaluate the expression of glycolysis-related genes revealed upregulation of the glycolytic markers PKM2 and PFKFB3 in CAFs and TGF-β-treated NIH-3T3 cells (Fig. 1I, J). These results indicate that CAFs have elevated glycolytic levels, leading to increased lactate production and subsequent acidification of the tumor microenvironment.

### Lactate enhances the stemness of OSCC cells

**Fig. 2:**
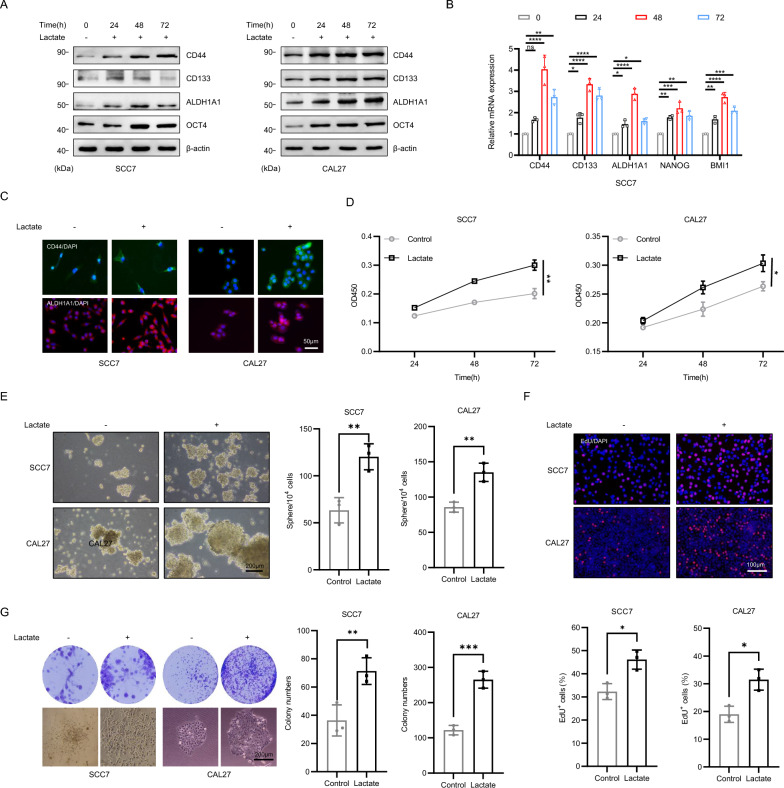
Lactate enhances the stemness of OSCC cells. **A**, **B** Western blotting and RT-qPCR results of CSCs-associated markers after exposure of SCC7 and CAL27 cells to 5.6 mM and 5.1 mM lactic acid concentrations for 0, 24, 48, and 72 h. β-actin and GAPDH were used as a control. **C** Cell immunofluorescence of CD44 and ALDH1A1 expression in lactate-treated tumor cells. Scale bars, 50 μm. **D** CCK8 assay was used to test cell viability. Student’s t-test. **E** Sphere formation assay was used to examine the sphere forming ability of lactate-treated cells. Scale bars, 200 μm. Student’s t-test. **F** EdU assay was used to test cell proliferation ability. Scale bars, 100 μm. Student’s t-test. **G** Colony formation assay was performed to examine the colony formation ability of lactate-treated cells. Student’s t-test. Scale bars, 200 μm. Independent experiments (in vitro) were performed in triplicate. Data are presented as mean±SD. *P < 0.05, **P < 0.01, ***P < 0.001, ****P < 0.0001

Studies have demonstrated a strong correlation between lactate and CSCs-like characteristics in tumor cells [33, 34]. To investigate the influence of lactate on the stem cell phenotype in oral squamous cell carcinoma, SCC7 and CAL27 cells were treated with lactate at concentrations of 5.6 mM and 5.1 mM, respectively, which matched the lactate levels detected in the supernatants of murine and human-derived CAF cultures. In addition, we collected tumor cell samples at 0, 24, 48, and 72 h following lactate treatment. Immunoblotting and PCR results indicated that the optimal effect of lactate on the tumor cells was achieved after 48h of exposure (Fig. 2A, B and Fig. S1A). Moreover, the fluorescence intensity of CD44 and ALDH1A1 was enhanced following lactate treatment (Fig. 2C). Compared with differentiated cancer cells, CSCs possess greater proliferation and self-renewal capacities [35, 36]. Both the EdU and CCK8 assays demonstrated that lactate promoted the proliferation of SCC7 and CAL27 cells, leading to a significant increase in the proportion of EdU-positive cells (Fig. 2D, F). Furthermore, compared with control cells, lactate-treated cancer cells exhibited enhanced spheroid and colony-forming abilities (Fig. 2E, G). These results indicate that lactate significantly enhances the CSCs phenotype in oral squamous cell carcinoma.

### Lactate upregulates DLG5 expression to promote the CSCs properties

**Fig. 3:**
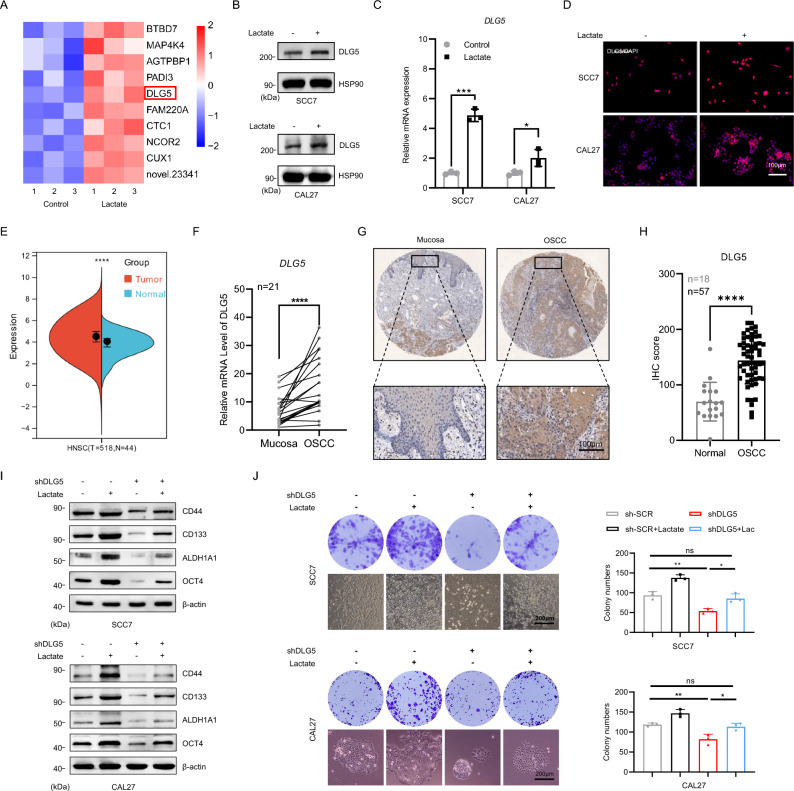
Lactate upregulates DLG5 expression to promote the CSCs properties. **A** Heat map of the top ten DEGs upregulated after lactate treatment of CAL27 cells. **C** represents the control group, and **D** represents the lactic acid treatment group. **B**–**D** Western blot, RT-qPCR and cell immunofluorescence results of DLG5 in SCC7 and CAL27 cells following exposed to lactate concentrations of 5.6 mM and 5.1 mM for 48 h, respectively. Scale bars, 100 μm. Student’s t-test. **E** The bioinformatics results obtained from SangerBox showed that DLG5 was highly expressed in tumors in HNSC. **F** DLG5 mRNA expression levels of paired normal mucosa and OSCC. Student’s t-test. **G**, **H** Immunohistochemical results and statistical analysis of DLG5 in normal mucosa and tumor tissues. Student’s t-test. Scale bars, 100 μm. **I** Western blot results of CSCs-related markers of sh-SCR group, sh-SCR+lactate treatment group, DLG5 knockdown group, lactate treatment+DLG5 knockdown group in tumor cells. β-actin was used as a control. **J** The colony formation experiment results and statistical results of the four groups of cells above were used to check the colony formation ability of the cells. One-way ANOVA. Scale bars, 200 μm. Independent experiments (*in vitro*) performed in triplicate. Data are presented as mean ± SD. *P < 0.05, **P < 0.01, ***P < 0.001, ****P < 0.0001

To elucidate the specific mechanisms by which lactate regulates the CSCs phenotype in OSCC, RNA sequencing (RNA-seq) analysis was performed on CAL27 cells treated with lactate. This analysis revealed 310 differentially expressed genes (DEGs), with DLG5 expression significantly upregulated following lactate treatment (Fig. 3A and Fig. S2A). The upregulation of DLG5 was confirmed through immunoblotting, PCR, and immunofluorescence assays (Fig. 3B–D). Considering the high glycolytic activity of CAFs, which results in an acidic tumor microenvironment, we investigated whether this could lead to increased expression of DLG5 in tumor tissues. Sangerbox online tool analysis demonstrated that DLG5 was highly expressed in head and neck squamous cell carcinoma (HNSCC) based on RNA-seq data from The Cancer Genome Atlas (TCGA) (Fig. 3E). In 21 paired clinical OSCC tissue samples, the mRNA level of DLG5 was greater in cancerous tissues than in adjacent normal oral epithelium (Fig. 3F). Immunohistochemistry results also indicated increased expression of DLG5 in OSCC tissues (Fig. 3G, H). Further analysis of tumor tissue samples revealed a positive correlation between DLG5 expression and both histopathological grade and clinical stage, independent of age, gender, or recurrence (Fig. S2B and supplemental file 4). Moreover, higher DLG5 expression was associated with a poorer patient prognosis (Fig. S2C). Furthermore, we used shRNA to knock down DLG5 expression in cancer cells (Fig. S3D). We observed that the impact of lactate on CSCs-related markers and the enhancement of clonogenic and sphere-forming abilities were significantly inhibited (Fig. 3I–J and Fig. S3E, F). To further demonstrate the effect of CAFs-derived lactate on tumor cells, we knocked down Monocarboxylate transporter 4 (MCT4), a key mediator of lactate transport across the plasma membrane, in CAFs and co-cultured them with tumor cells, with or without DLG5 knockdown (Fig. S3G) [37]. The experimental results were consistent with the previous findings (Fig. S3H–J), suggesting that knockdown of DLG5 inhibited the lactate-driven enhancement of stemness markers.

### DLG5 inactivating Hippo pathway through inhibiting CUL3-mediated degradation of MST1

**Fig. 4:**
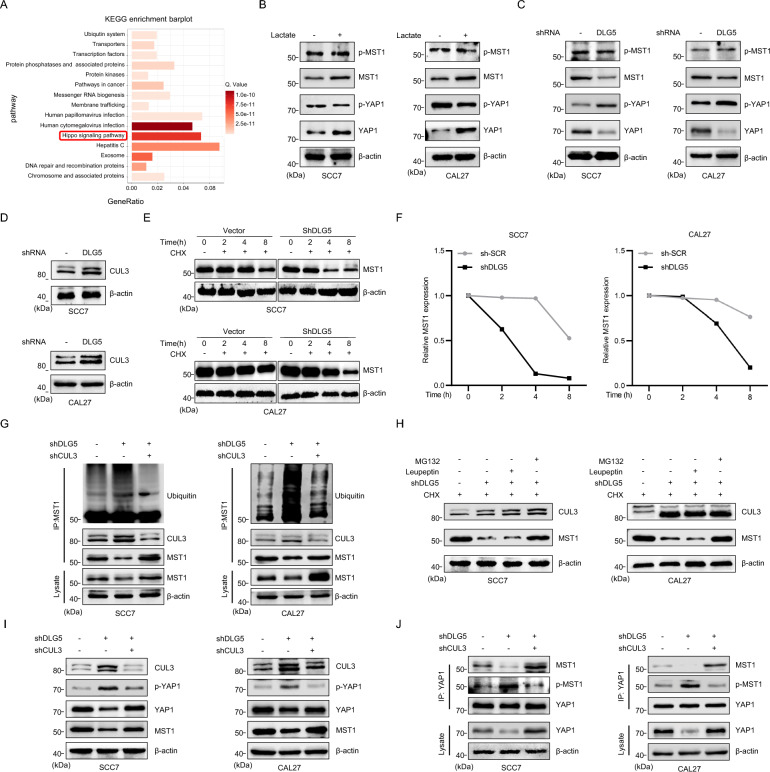
DLG5 inactivating Hippo pathway through inhibiting CUL3-mediated degradation of MST1. **A** Kyoto Encyclopedia of Genes and Genomes (KEGG) obtained from RNA-seq results of tumor cells following exposed to lactate concentrations of 5.6 mM and 5.1 mM for 48 h, respectively. **B**, **C** Western blot results of Hippo pathway related indicators in tumor cells after lactate stimulation and DLG5 knockdown. β-actin was used as a control. **D** Western blot results of Cul3 in tumor cells after DLG5 knockdown. β-actin was used as a control. **E**, **F** Western blot results and quantitative analysis of MST1 in sh-SCR group and DLG5 knockdown group cells treated with CHX (protein synthesis inhibitors) at a concentration of 100 μM for 0, 2, 4, and 8 h, respectively. β-actin was used as a control. **G** Anti-Ub immunoblotting assay of MST1 polyubiquitination in SCC7 and CAL27 cells after knockdown DLG5 or Cul3. **H **Western blot analysis of MST1, Cul3 and β-actin proteins in tumor cells knocked down by DLG5 after 8 h of CHX (100 μM) stimulation. At the same time, add MG132 (50 μM) or Leupeptin (50 μM). **I** Western blot results of Hippo pathway related indicators in tumor cells after Cul3 and DLG5 knockdown. β-actin was used as a control. **J** Co-Immunoprecipitation assay for detecting the binding status of YAP1 with p-MST1 and MST1. To clarify the binding status of YAP1 with p-MST1 and MST1, we used an equal but relatively small amount of YAP1 antibody mixed with magnetic bead cell lysis solution for pull-down. β-actin was used as a control

What is the specific mechanism by which DLG5 affects the CSC phenotype? Analysis of DEGs following lactate treatment revealed enrichment in the Hippo pathway (Fig. 4A). YAP1, a transcriptional coactivator downstream of the Hippo pathway, is strictly regulated by mammalian sterile 20-like kinase (MST) 1/2 and large tumor suppressors1/2 (LATS1/2) [21, 38, 39]. Immunoblotting showed that lactate treatment led to a decrease in phosphorylated YAP1 (p-YAP1) and an increase in YAP1 expression, indicating inactivation of the Hippo–YAP1 pathway. Furthermore, although phosphorylated MST1 (p-MST1) levels remained unchanged, total MST1 expression increased (Fig. 4B). To confirm the role of DLG5 in regulating the Hippo–YAP1 pathway, we knocked down DLG5 and observed opposing effects on Hippo–YAP1 pathway-related proteins compared to those of lactate treatment, with increased p-YAP1 and decreased YAP1 expression (Fig. 4C). And it was observed that the addition of lactate counteracted the HIPPO pathway activation effect induced by knocking down DLG5 (Fig. S3A). Furthermore, we found that the addition of lactate repressed the HIPPO pathway, thereby promoting the expression of downstream transcription factors CTGF, BIRC5, and TOP2A [40], while knockdown of DLG5 had the opposite effect (Fig. S3B-C). Research suggests that DLG5 regulates the expression of the E3 ubiquitin ligase CUL3 [32]. The immunoblotting results demonstrated that knocking down DLG5 increased CUL3 expression in SCC7 and CAL27 cells (Fig. 4D), accelerating MST1 degradation (Fig. 4E, F). Coimmunoprecipitation (Co-IP) indicated that CUL3 binds to MST1 but not to p-MST1 (Fig. S3E). Knockdown of DLG5 increased the level of ubiquitination of MST1, whereas simultaneous knockdown of CUL3 inhibited this ubiquitination (Fig. 4G and Fig. S3D, F). Notably, the proteasome inhibitor MG132 effectively blocked MST1 degradation, whereas the lysosome inhibitor leupeptin had no significant effect (Fig. 4H). These findings suggest that DLG5 downregulates CUL3, reducing MST1 ubiquitination and subsequent proteasomal degradation. Interestingly, whereas CUL3 promotes the ubiquitin-mediated degradation of MST1, it does not significantly affect the levels of p-MST1, which plays a crucial role in YAP1 phosphorylation. Further studies revealed that nonphosphorylated MST1 competes with p-MST1 for binding to YAP1. Following DLG5 knockdown, increased degradation of MST1 allowed more p-MST1 to bind to YAP1, leading to increased YAP1 phosphorylation, whereas knocking down CUL3 reversed this change (Fig. 4I, J and Fig. S3G). In summary, these results demonstrate that DLG5, by downregulating CUL3, inhibits MST1 ubiquitination and degradation, inducing YAP1 dephosphorylation and inactivation of the Hippo–YAP1 pathway.

### Lactate promote the progression of OSCC in vivo

**Fig. 5:**
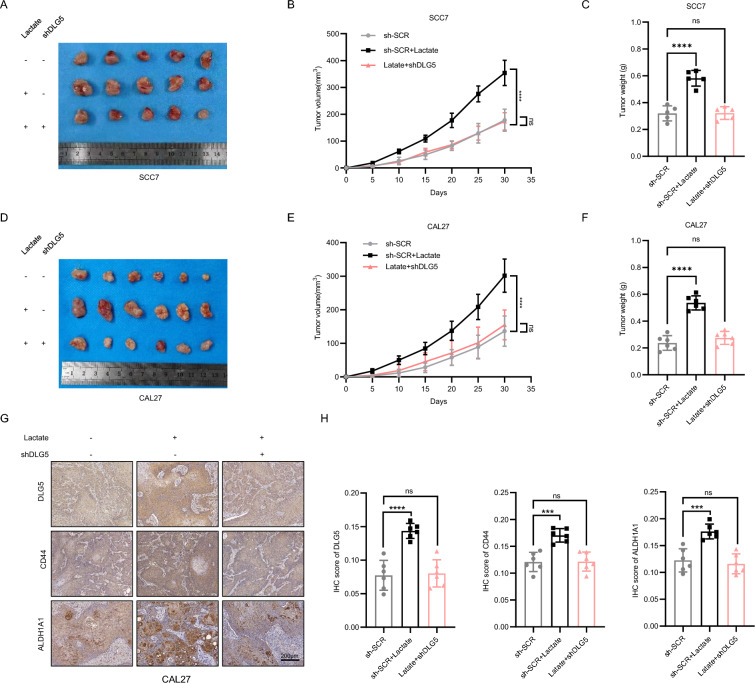
Lactate promote the progression of OSCC in vivo. **A** Images of three xenograft tumors of SCC7 after lactate treatment or knocking down DLG5. **B**, **C** The tumor volume growth curve and weight of the subcutaneous tumorigenic model (SCC7). One-way ANOVA. **D** Images of three xenograft tumors of CAL27 after lactate treatment or knocking down DLG5. **E**, **F** The tumor volume growth curve and weight of the subcutaneous tumorigenic model (CAL27). One-way ANOVA. **G** Representative IHC images of DLG5, CD44, and ALDH1A1 in three groups in xenograft tumors (CAL27). Scale bars, 200μm. One-way ANOVA. **H** Relative IHC score of DLG5, CD44, and ALDH1A1 (CAL27). One-way ANOVA. Data are presented as mean ± SD. *P < 0.05, **P < 0.01, ***P < 0.001, ****P < 0.0001

To further elucidate the role of the lactate-DLG5 axis in the progression of OSCC, in vivo tumorigenicity assays were conducted using SCC7 and CAL27 cells. The results of the present study demonstrated that compared with those in the sh-SCR group, the weights and volumes of the tumors in the sh-SCR + lactate treated group were significantly greater. However, this effect was inhibited by the knockdown of DLG5 (Fig. 5A–F). Immunohistochemical staining also revealed an increase in the expression of DLG5, CD44, and ALDH1A1 in the sh-SCR + lactate treated group, whereas the knockdown of DLG5 resulted in a reversal of stem cell marker expression (Fig. 5G, H and Fig. S2A, B). These findings provide strong evidence that lactate promotes the progression of OSCC and that the knockdown of DLG5 effectively prevents the lactate-induced enhancement of the OSCC CSCs phenotype.

## Discussion

**Fig. 6:**
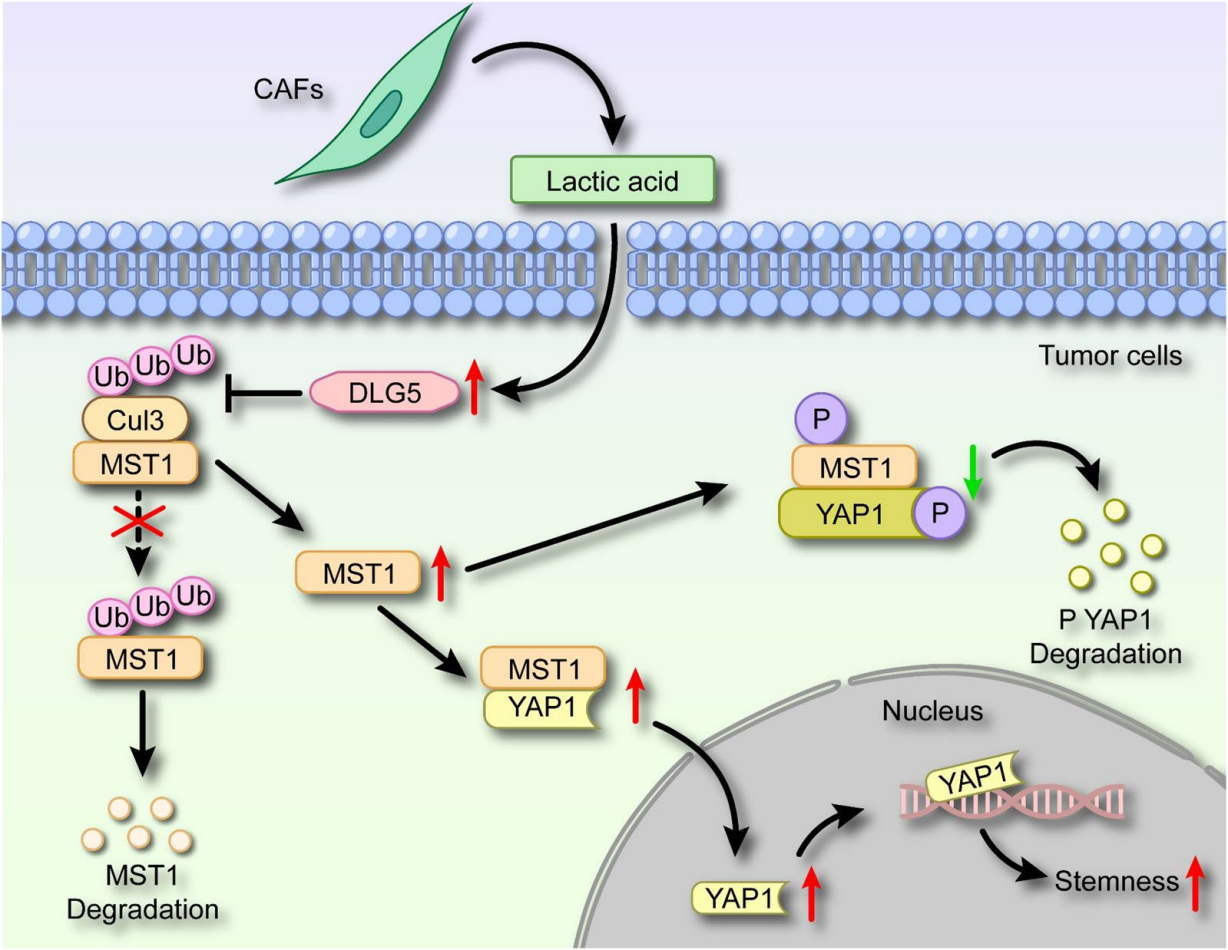
Schematic diagram of CAFs-derived lactate in regulating the CSCs properties of OSCC. Lactate derived from CAFs upregulates the expression of DLG5 in tumor cells, interferes with CUL3 binding to MST1, inhibits the ubiquitination degradation of MST1, inactivates the HIPPO pathway, promotes YAP1 nuclear localization, and enhances the phenotype of tumor stem cells

CSCs are a subpopulation of cells that are closely associated with tumor progression, metastasis, and drug resistance. CSCs may also play a significant role in OSCC, and understanding the molecular mechanisms that regulate CSCs could improve the prognosis of patients with OSCC [41–44]. In this study, we made the important discovery that CAFs in OSCC exhibit elevated levels of glycolysis, resulting in increased production of lactate. Further investigation revealed that lactate derived from CAFs negatively regulates the HIPPO pathway through the DLG5/CUL3/MST1 axis, thus promoting the nuclear localization of YAP1 and enhancing the tumor stem cell phenotype (Fig. 6). These findings provide valuable insights into the intrinsic mechanism by which lactate in the tumor microenvironment influences the CSCs phenotype, suggesting that targeting the transport of lactate between CAFs and tumor cells could be a promising therapeutic approach for OSCC.

CAFs play a critical role in the tumor microenvironment and are closely associated with an unfavorable prognosis in oral squamous cell carcinoma [45, 46]. CAFs control various aspects of tumor development, including cell survival, proliferation, angiogenesis, extracellular matrix modification, metastasis, and resistance to chemotherapy. According to the reverse Warburg effect, CAFs primarily rely on aerobic glycolysis, generating significant amounts of lactate that cancer cells take up and utilize for oxidative phosphorylation, thereby facilitating rapid tumor growth. A study conducted by Zhang et al. demonstrated that CAFs expressing ITGβ2 enhance glycolytic activity through the PI3K/AKT/mTOR pathway, resulting in the secretion of lactate, which promotes the proliferation of OSCC cells [47]. In our own research, we observed that compared with PFs, CAFs exhibit higher levels of glycolysis, leading to increased lactate production. Additionally, CSCs, a minor subpopulation of cancer cells distinguished by their self-renewal, regenerative, and differentiating abilities, play a pivotal role in tumor heterogeneity [48, 49]. Previous coculture experiments involving CAFs and oral squamous cell carcinoma cells in 3D organoids revealed that the lactate produced by CAFs promotes the formation of OSCC organoids [10, 20, 50]. In our current study, we confirmed that the addition of lactate increases the expression of CSCs markers, as well as the sphere and colony formation capabilities, thereby confirming that lactate promotes the CSCs phenotype in oral squamous cell carcinoma.

DLG5 possesses four PDZ domains, one SH3 domain, and one GUK domain, which play crucial roles in the maintenance of epithelial cell polarity and cancer progression [51]. Our research revealed a significant increase in DLG5 expression in SCC7 and CAL27 cells upon exposure to lactate. Recent studies have also shown that DLG5 can bind directly to MST1/2 through its third PDZ domain, resulting in the negative regulation of the Hippo pathway [52]. The Hippo pathway is an essential signaling cascade that is closely associated with the maintenance of the tumor stem cell phenotype when inactivated. MST1/2 are core kinases upstream of this pathway, and various cellular signals induce their phosphorylation, leading to the degradation of YAP1 and the activation of the Hippo pathway [53]. Interestingly, our findings revealed that both phosphorylated and unphosphorylated MST1 can bind to YAP1. Knocking down DLG5 in our experiments resulted in a decrease in MST1 expression without affecting the levels of phosphorylated MST1. This led to a relative increase in the availability of phosphorylated MST1 to bind to YAP1, promoting the phosphorylation and degradation of YAP1 and inhibiting its nuclear localization. Ubiquitination, a common posttranslational modification of proteins, plays a fundamental role in regulating protein half-life, localization, and activity [54]. Our study showed that DLG5 reduces the expression of CUL3, which in turn decreases the ubiquitin-mediated degradation of MST1. Additionally, the knockdown of DLG5 inhibited the CSCs phenotype in oral squamous cell carcinoma. However, our research has certain limitations. First, while we employed both human- and murine-derived CAFs and tumor cells, our primary findings were based on OSCC cell lines and tumorigenic models in nude mice, which may not fully represent the in vivo conditions. Second, although lactylation research has been progressing rapidly and is a current focus in the field, this paper does not explore in depth whether lactate’s regulation of the CSC phenotype is primarily mediated through lactylation [55, 56]. In the future, it would be beneficial to conduct further experiments using DLG5 conditional knockout mice or more advanced models, such as patient-derived organoids or patient-derived xenografts. And it is crucial for us to investigate whether the regulation of CSC phenotype by lactate is largely dependent on lactylation. In conclusion, our study revealed that lactate derived from cancer-associated fibroblasts promotes the CSCs phenotype in oral squamous cell carcinoma through the DLG5/CUL3/MST1 signaling axis. Targeting the lactate shuttle between cancer-associated fibroblasts and tumor cells could be an effective therapeutic strategy to suppress the CSCs phenotype in oral squamous cell carcinoma, representing a novel therapeutic target.

## Supplementary Information

Additional file 1.

Additional file 2.

Additional file 3.

Additional file 4.

Additional file 5.

## Data Availability

All data generated or analyzed in this study are included in this published article and its supplementary information files.

## Declarations

### Animal studies

Animal experiments were approved by the Ethical Committee on Animal Experiments of the Animal Care Committee of Wuhan University, approved number: S0792203066.

Informed Consent: Informed consent was obtained from all subjects for tissue sample collection.

### Registry and the registration No. of the study

Ethics Committee of School and Hospital of Stomatology, Wuhan University, 2022-A05.
