# Supplementary material for: CAFs-derived lactate enhances the cancer stemness through inhibiting the MST1 ubiquitination degradation in OSCC
Source: Cell Biosci. 2024 Nov 27;14:144. doi: 10.1186/s13578-024-01329-y (PMC11603751; doi:10.1186/s13578-024-01329-y)
Supplement: Supplementary file 1 — Additional file 1. [file 13578_2024_1329_MOESM1_ESM.docx]

**CAFs-derived lactate enhances the cancer stemness through inhibiting the MST1 ubiquitination degradation in OSCC**

Authors’ names: Shuzhen Zhang^1, 7, #^, Jingjing Wang^1, #^, Yang Chen^1, 3, #^, Hanzhe Liu^1^, Ruixue Du^1^, Yunqing Sun^1^, Chuanyu Hu^4,5,6 *^, Zhengjun Shang^1, 2, *^

^1^ State Key Laboratory of Oral & Maxillofacial Reconstruction and Regeneration, Key Laboratory of Oral Biomedicine Ministry of Education, Hubei Key Laboratory of Stomatology, School & Hospital of Stomatology, Wuhan University.

^2^ Department of Oral and Maxillofacial-Head and Neck Oncology, School & Hospital of Stomatology, Wuhan University, Wuhan 430079, China.

^3^ Department of Oral and Maxillofacial Surgery, School & Hospital of Stomatology, Wuhan University, Wuhan 430079, China.

^4^Department of Stomatology, Tongji Hospital, Tongji Medical College, Huazhong University of Science and Technology, Wuhan, China

^5^School of Stomatology, Tongji Medical College, Huazhong University of Science and Technology, Wuhan, China

^6^Hubei Province Key Laboratory of Oral and Maxillofacial Development and Regeneration, Wuhan, China

^7^Department of The third out-patient，School & Hospital of Stomatology, Wuhan University, Wuhan 430022, China.

**# Shuzhen Zhang, Jingjing Wang and Yang Chen contributed equally to this study.**

*******Corresponding authors:** Chuanyu Hu, Department of Stomatology, Tongji Hospital, Tongji Medical College, Huazhong University of Science and Technology, Wuhan 430030, China. Email: chuanyuhu@hust.edu.cn

Zhengjun Shang, The State Key Laboratory Breeding Base of Basic Science of Stomatology, Hubei Province & Key Laboratory of Oral Biomedicine (Wuhan University), Ministry of Education (Hubei-MOST KLOS & KLOBM), Wuhan 430079, China. Email: [shangzhengjun@whu.edu.cn](mailto:shangzhengjun@whu.edu.cn).

**Methods and materials**

**pH measurement**

PFs and CAFs were seeded into the six-well plate at 30-40% cell density, and an equal amount of culture medium was added. After 48h, the supernatant was collected and the pH value of the culture medium was measured using a pH meter (Bohlertech Technology, China).

**Glucose consumption experiments**

Seeding equal amounts of cells into a 6-well plate, wait until the cell density reaches about 90%, digest the cells with trypsin, centrifuge to discard the supernatant, and add 200μl distilled water, sonicated at 95℃ for 10 min, cooled to 8000g, centrifuged at 25℃ for 10 min, and the supernatant was retained. Follow the instructions of the Glucose content kit (BC2500, Solarbio, China) for the subsequent steps, read the absorbance at a wavelength of 505nm and record it.

**Immunohistochemistry**

The protein expressions of DLG5, CD44, and ALDH1A1 in tissue samples were detected through immunohistochemical (IHC) staining. The tumor tissue was fixed with formalin, embedded in paraffin, sliced to 4μm thickness, and then mounted on a slide. The protein expression of transplanted tumor sections was determined using immunohistochemical staining. Staining was performed utilizing the anti-rabbit or anti-mouse UltraSensitiveTM SP kit (Maixin, Fuzhou, China) following the manufacturer's protocol. After deparaffinization and rehydration steps, antigen retrieval on the sections was achieved by boiling in 10mM sodium citrate buffer (pH 6.0) for 10 min. Endogenous peroxidase activity was inhibited by incubating at room temperature with 3% H_2_O_2_ for 20 min. Following three washes with PBS, the slides were blocked with 100µL of normal goat serum at room temperature for 30 min and kept for an additional hour. Subsequently, the slides were incubated overnight at 4°C with primary antibodies (DLG5, CD44, and ALDH1A1), followed by incubation with secondary antibodies at room temperature for 1h. The stained slide was visualized using diaminobenzidine as a color-developing substrate and counterstained with hematoxylin. A negative control (no primary antibody culture) was included during each step of immunostaining process. Immunohistochemical assessments were conducted according to previously published protocols.
