# Supplementary material for: CAFs-derived lactate enhances the cancer stemness through inhibiting the MST1 ubiquitination degradation in OSCC
Source: Cell Biosci. 2024 Nov 27;14:144. doi: 10.1186/s13578-024-01329-y (PMC11603751; doi:10.1186/s13578-024-01329-y)
Supplement: Supplementary file 2 — Additional file 2. [file 13578_2024_1329_MOESM2_ESM.docx]

**CAFs-derived lactate enhances the cancer stemness through inhibiting the MST1 ubiquitination degradation in OSCC**

Authors’ names: Shuzhen Zhang^1, 7, #^, Jingjing Wang^1, #^, Yang Chen^1, 3, #^, Hanzhe Liu^1^, Ruixue Du^1^, Yunqing Sun^1^, Chuanyu Hu^4,5,6 *^, Zhengjun Shang^1, 2, *^

^1^ State Key Laboratory of Oral & Maxillofacial Reconstruction and Regeneration, Key Laboratory of Oral Biomedicine Ministry of Education, Hubei Key Laboratory of Stomatology, School & Hospital of Stomatology, Wuhan University.

^2^ Department of Oral and Maxillofacial-Head and Neck Oncology, School & Hospital of Stomatology, Wuhan University, Wuhan 430079, China.

^3^ Department of Oral and Maxillofacial Surgery, School & Hospital of Stomatology, Wuhan University, Wuhan 430079, China.

^4^Department of Stomatology, Tongji Hospital, Tongji Medical College, Huazhong University of Science and Technology, Wuhan, China

^5^School of Stomatology, Tongji Medical College, Huazhong University of Science and Technology, Wuhan, China

^6^Hubei Province Key Laboratory of Oral and Maxillofacial Development and Regeneration, Wuhan, China

^7^Department of The third out-patient，School & Hospital of Stomatology, Wuhan University, Wuhan 430022, China.

**# Shuzhen Zhang, Jingjing Wang and Yang Chen contributed equally to this study.**

*******Corresponding authors:** Chuanyu Hu, Department of Stomatology, Tongji Hospital, Tongji Medical College, Huazhong University of Science and Technology, Wuhan 430030, China. Email: chuanyuhu@hust.edu.cn

Zhengjun Shang, The State Key Laboratory Breeding Base of Basic Science of Stomatology, Hubei Province & Key Laboratory of Oral Biomedicine (Wuhan University), Ministry of Education (Hubei-MOST KLOS & KLOBM), Wuhan 430079, China. Email: [shangzhengjun@whu.edu.cn](mailto:shangzhengjun@whu.edu.cn).

**
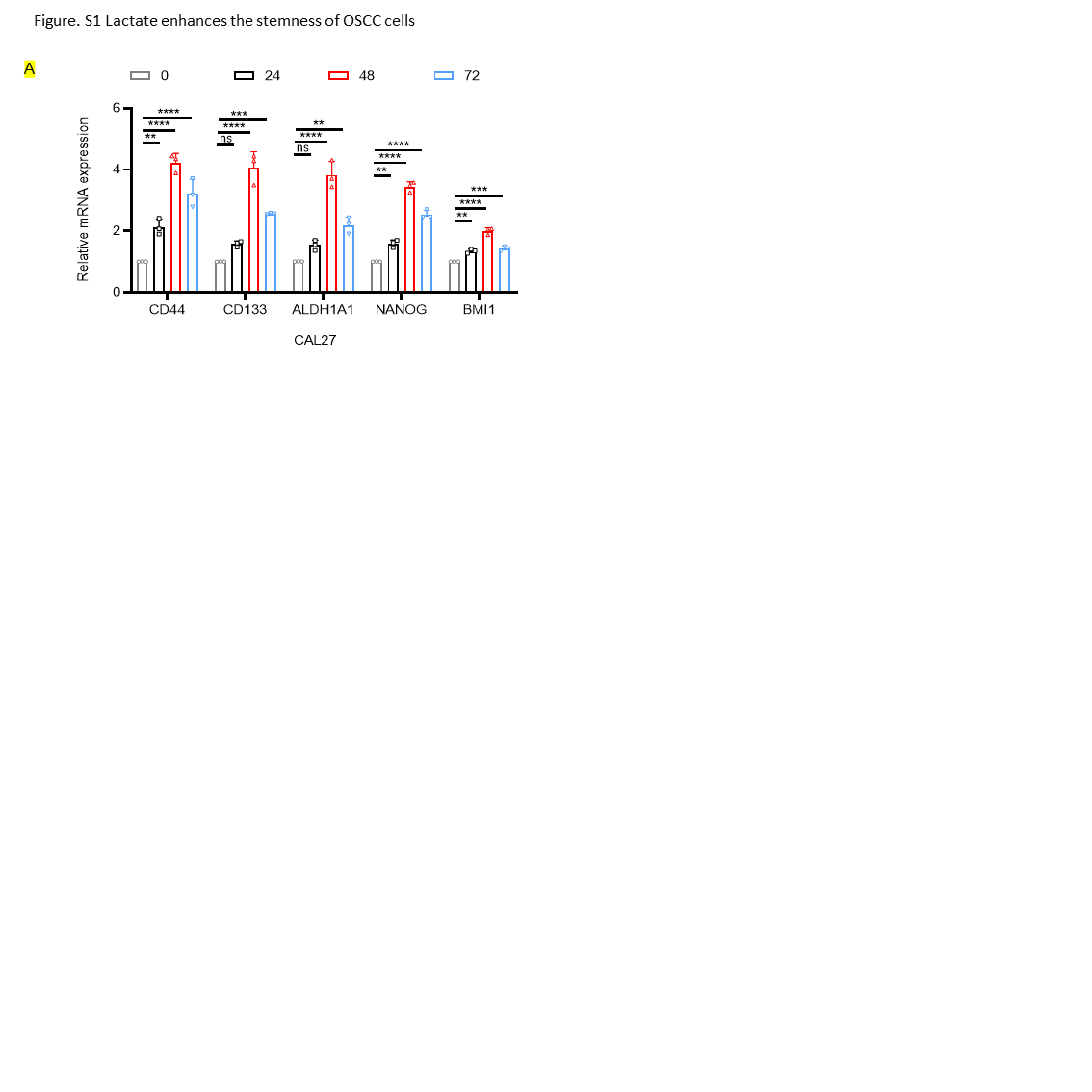
Supplement Figures**

**Figure. S1 Lactate enhances the stemness of OSCC cells**

**(A)** RT-qPCR results of stemness markers in tumor cells after stimulation with lactate for 0, 24, 48, and 72 hours. β-actin and GAPDH were used as a control.

**
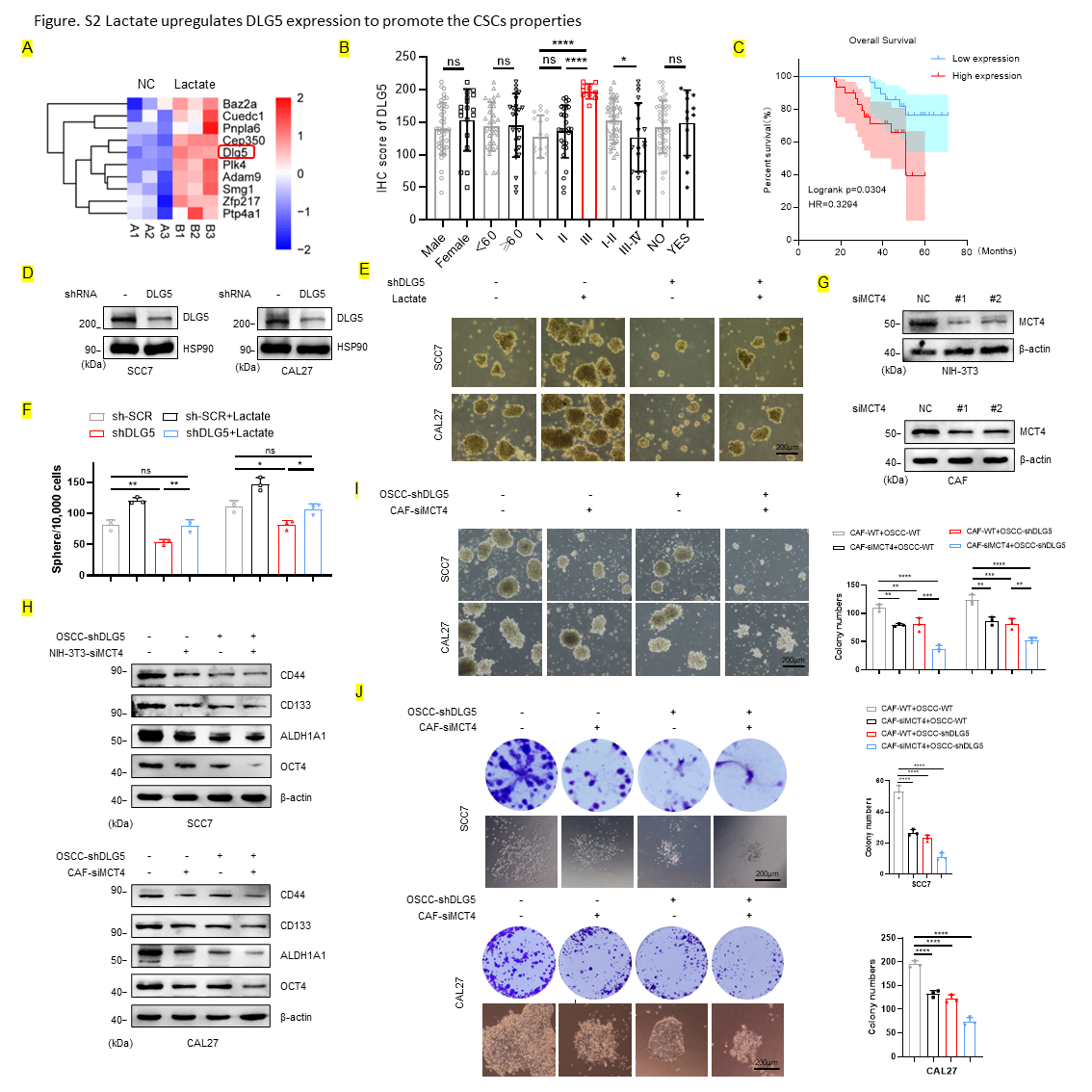
Figure. S2 Lactate upregulates DLG5 expression to promote the CSCs properties**

**(A)** Heat map of the top ten DEGs upregulated after lactate treatment of SCC7 cells. A represents the control group, and B represents the lactic acid treatment group. **(B)** The expression of DLG5 in OSCC tissues at different clinical stages, recurrence statuses, pathological grades, ages, and genders. n=57. **(C)** Overall survival based on DLG5 expression in OSCC. n=57. **(D)** Western blot results of DLG5 in tumor cells after DLG5 knockdown. HSP90 was used as a control. **(E, F)** The results of the four groups (sh-SCR group, sh-SCR+lactate treatment group, DLG5 knockdown group, lactate treatment+DLG5 knockdown group) of sphere formation assay and statistical analysis were used to examine the self-renewal ability of cells. One-way ANOVA. Scale bars, 200μm. **(G)** Western blot was used to assess the knockdown efficiency of MCT4 in CAFs or NIH-3T3(TGF-β) by two different siRNAs. β-actin was used as a control. **(H)** After knocking down MCT4 in CAFs or NIH-3T3(TGF-β), they were co-cultured with tumor cells, with or without DLG5 knockdown, for 48 hours. The results of the Western blot for stemness markers in tumor cells were then analyzed. β-actin was used as a control. **(I)** The sphere formation assay results and statistical analysis results of the four groups of cells above were used to examine the self-renewal ability of cells. One-way ANOVA. Scale bars, 200μm. **(J)** The colony formation experiment results and statistical results of the four groups of cells above were used to check the colony formation ability of the cells. One-way ANOVA. Scale bars, 200μm. `Independent experiments (*in vitro*) performed in triplicate. Data are presented as mean±SD. *, *P* < 0.05, **, *P* < 0.01, ***, *P* < 0.001, ****, *P* < 0.0001.

**
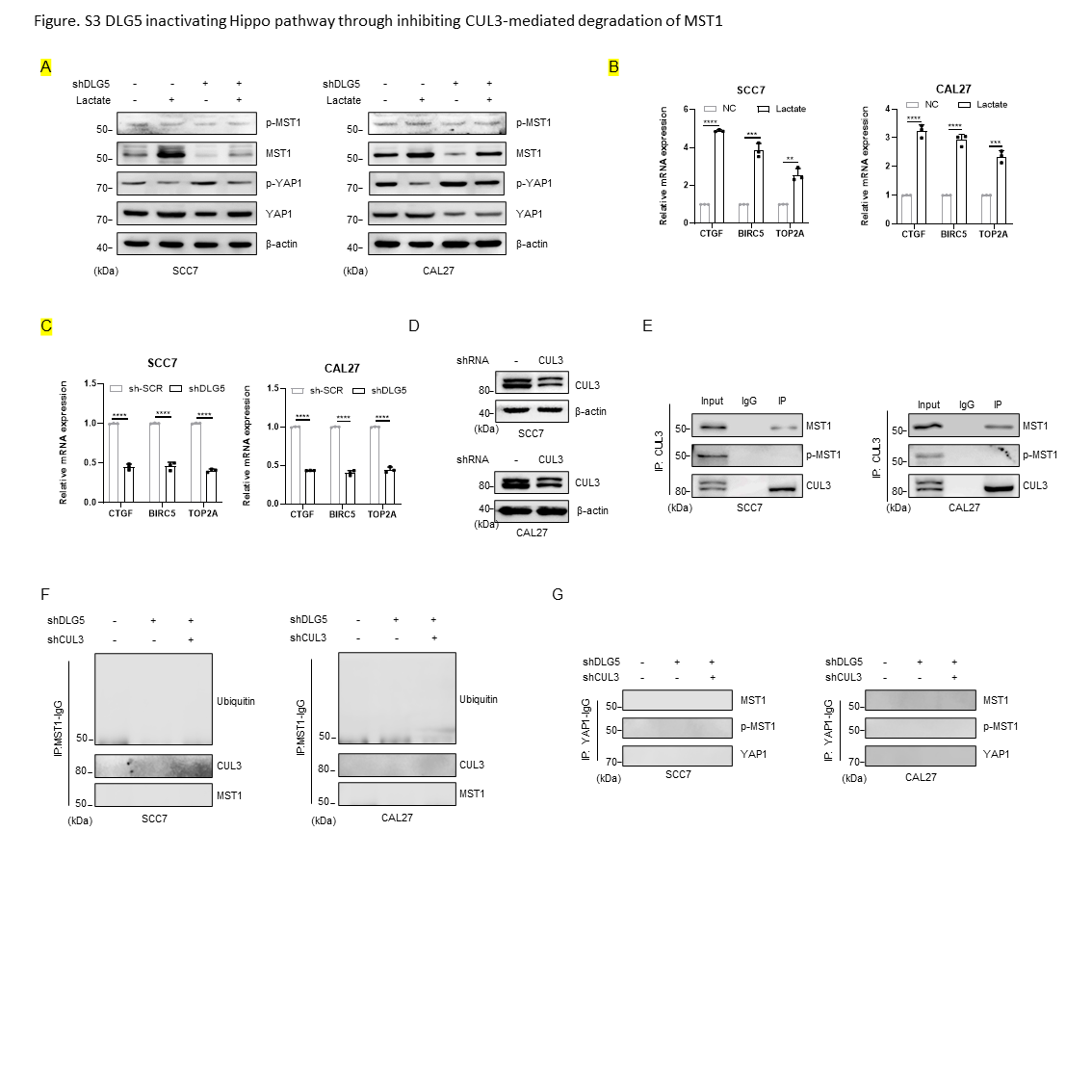
Figure. S3 DLG5 inactivating Hippo pathway through inhibiting CUL3-mediated degradation of MST1**

**(A)** Western blot results of Hippo pathway related indicators of sh-SCR group, sh-SCR+lactate treatment group, DLG5 knockdown group, lactate treatment+DLG5 knockdown group in tumor cells. β-actin was used as a control. **(B)** RT-qPCR results of CTGF, BIRC5 and TOP2A in tumor cells after lactate stimulation. GAPDH was used as a control. **(C)** RT-qPCR results of CTGF, BIRC5 and TOP2A in tumor cells after DLG5 knockdown. GAPDH was used as a control. **(D)** Western blot results of Cul3 in tumor cells after Cul3 knockdown. β-actin was used as a control. **(E)** Co-Immunoprecipitation assay for detecting the binding status of Cul3 with p-MST1 and MST1 in SCC7 and CAL27. **(F)** Anti-Ub immunoblotting assay of MST1 polyubiquitination in SCC7 and CAL27 cells after knockdown DLG5 or Cul3. **(G)** Co-Immunoprecipitation assay for detecting the binding status of YAP1 with p-MST1 and MST1. To clarify the binding status of YAP1 with p-MST1 and MST1, we used an equal but relatively small amount of YAP1 antibody mixed with magnetic bead cell lysis solution for pull-down. β-actin was used as a control.

**
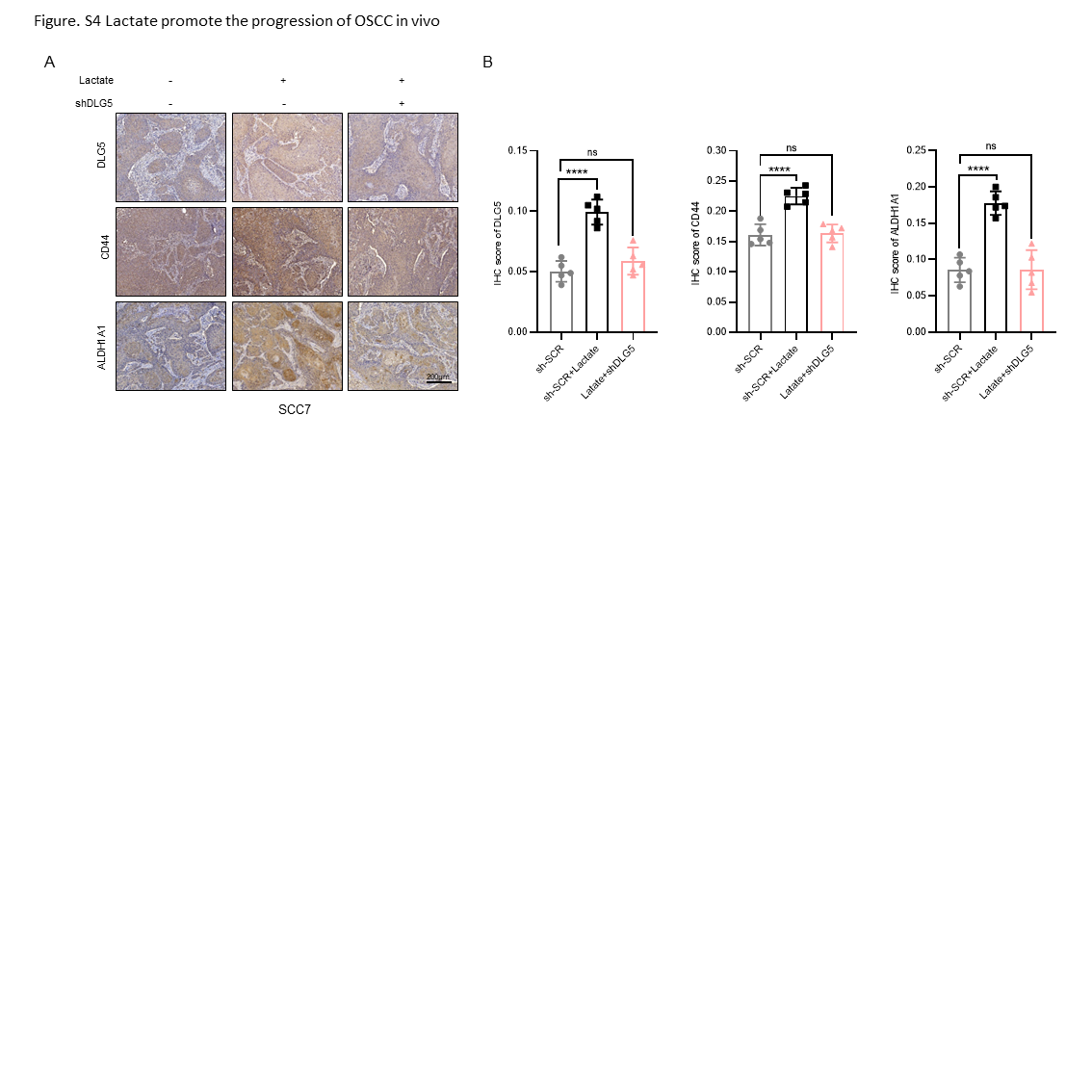
Figure. S4 Lactate promote the progression of OSCC in vivo**

**(A)** Representative IHC images of DLG5, CD44, and ALDH1A1 in three groups in xenograft tumors (SCC7). Scale bars, 200μm. One-way ANOVA. **(B)** Relative IHC score of DLG5, CD44, and ALDH1A1 (SCC7). One-way ANOVA. Data are presented as mean±SD. *, *P* < 0.05, **, *P* < 0.01, ***, *P* < 0.001, ****, *P* < 0.0001.
