# Supplementary material for: CAFs-derived lactate enhances the cancer stemness through inhibiting the MST1 ubiquitination degradation in OSCC
Source: Cell Biosci. 2024 Nov 27;14:144. doi: 10.1186/s13578-024-01329-y (PMC11603751; doi:10.1186/s13578-024-01329-y)
Supplement: Supplementary file 3 — Additional file 3. [file 13578_2024_1329_MOESM3_ESM.docx]

**CAFs-derived lactate enhances the cancer stemness through inhibiting the MST1 ubiquitination degradation in OSCC**

Authors’ names: Shuzhen Zhang^1, 7, #^, Jingjing Wang^1, #^, Yang Chen^1, 3, #^, Hanzhe Liu^1^, Ruixue Du^1^, Yunqing Sun^1^, Chuanyu Hu^4,5,6 *^, Zhengjun Shang^1, 2, *^

^1^ State Key Laboratory of Oral & Maxillofacial Reconstruction and Regeneration, Key Laboratory of Oral Biomedicine Ministry of Education, Hubei Key Laboratory of Stomatology, School & Hospital of Stomatology, Wuhan University.

^2^ Department of Oral and Maxillofacial-Head and Neck Oncology, School & Hospital of Stomatology, Wuhan University, Wuhan 430079, China.

^3^ Department of Oral and Maxillofacial Surgery, School & Hospital of Stomatology, Wuhan University, Wuhan 430079, China.

^4^Department of Stomatology, Tongji Hospital, Tongji Medical College, Huazhong University of Science and Technology, Wuhan, China

^5^School of Stomatology, Tongji Medical College, Huazhong University of Science and Technology, Wuhan, China

^6^Hubei Province Key Laboratory of Oral and Maxillofacial Development and Regeneration, Wuhan, China

^7^Department of The third out-patient，School & Hospital of Stomatology, Wuhan University, Wuhan 430022, China.

**# Shuzhen Zhang, Jingjing Wang and Yang Chen contributed equally to this study.**

*******Corresponding authors:** Chuanyu Hu, Department of Stomatology, Tongji Hospital, Tongji Medical College, Huazhong University of Science and Technology, Wuhan 430030, China. Email: chuanyuhu@hust.edu.cn

Zhengjun Shang, The State Key Laboratory Breeding Base of Basic Science of Stomatology, Hubei Province & Key Laboratory of Oral Biomedicine (Wuhan University), Ministry of Education (Hubei-MOST KLOS & KLOBM), Wuhan 430079, China. Email: [shangzhengjun@whu.edu.cn](mailto:shangzhengjun@whu.edu.cn).

**Table S1.**

**Primers used in the study.**

| Primers | Sequences（5'-3'） |
| --- | --- |
| CD44 forward primer | CTGCCGCTTTGCAGGTGTA |
| CD44 reverse primer | CATTGTGGGCAAGGTGCTATT |
| CD133 forward primer  CD133 reverse primer  ALDH1A1 forward primer  ALDH1A1 reverse primer  GAPDH forward primer  GAPDH reverse primer  DLG5 forward primer  DLG5 reverse primer  NRF2 forward primer  NRF2 reverse primer  PGC1α forward primer  PGC1α reverse primer  CTGF forward primer  CTGF reverse primer  BIRC5 forward primer  BIRC5 reverse primer  TOP2A forward primer  TOP2A reverse primer | AGTCGGAAACTGGCAGATAGC GGTAGTGTTGTACTGGGCCAAT  CCGTGGCGTACTATGGATGC  GCAGCAGACGATCTCTTTCGAT  ACAACTTTGGTATCGTGGAAGG  GCCATCACGCCACAGTTTC  TGAGGCGATCCACCATGAG  CCTCCCTGTATTTCTCCGACT  TCCAGTCAGAAACCAGTGGAT  GAATGTCTGCGCCAAAAGCTG  TCTGAGTCTGTATGGAGTGACAT  CCAAGTCGTTCACATCTAGTTCA  CACCACCACCAAGTCCTACC  CTTCTTCATGACCTGGCCGT  GACTTCTGTTGCCTTGAAATGA  ATGTCCTGTTGCACTAAGGGA  TGCCAAAACCAAGAATCGCC  TCAACACTCACCTTGCTTGTG |

**Table S2**

**The sequences of siRNAs.**

| Oligonucleotides | Sequences |
| --- | --- |
| siMCT4-1  siMCT4-1 | 5’-3’ CCAGACATACAATGGCTCTTUTT  3’-5’ AAAAAGAGCCATTGTATGTCTGG |
| siMCT4-2 | 5’-3’ GCCACCACATTTCCACTACTUTT |
| siMCT4-2  siMct4-1  siMct4-1  siMct4-2  siMct4-2  sh-SCR  shDLG5  shDlg5  shCUL3  shCul3 | 3’-5’ AAAAGTAGTGGAAATGTGGTGGC  5’-3’ ACAGACTTACACAGGCTCTTUTT  3’-5’ AAAAAGAGCCTGTGTAAGTCTGT  5’-3’ GCTCTTTAGAAACCCTTTCTUTT  3’-5’ AAAAGAAAGGGTTTCTAAAGAGC  5’-3’ UUCUCCGAACGUGUCACGUTT  5’-3’ CCTGGGTTCTTCGAGTAACTT  5’-3’ GCATTGCTGATGGTCGCTTAA  5’-3’ CGTAGACAGAGGCGCAATAAG  5’-3’ AGCTGCTATAGTGCGAATAAT |
